# Exergoeconomic and Thermodynamic Analyses of Solar Power Tower Based Novel Combined Helium Brayton Cycle‐Transcritical CO_2_ Cycle for Carbon Free Power Generation

**DOI:** 10.1002/gch2.202300191

**Published:** 2023-11-03

**Authors:** Yunis Khan, Deepak Singh, Hakan Caliskan, Hiki Hong

**Affiliations:** ^1^ Department of Mechanical Engineering Delhi Technological University Delhi 110042 India; ^2^ Department of Mechanical Engineering Lokmanya Tilak College of Engineering Sector‐04, Vikas Nagar, Koparkhairne Navi Mumbai 400709 India; ^3^ Department of Mechanical Engineering Faculty of Engineering and Natural Science Usak University Usak 64200 Turkey; ^4^ Department of Mechanical Engineering Kyung Hee University Yongin 17104 Republic of Korea

**Keywords:** exergoeconomic analysis, Exergy analysis, helium Brayton cycle, solar power tower, transcritical CO_2_ cycle

## Abstract

In the present study, a novel combined power cycle for solar power tower (SPT) system consisting of helium Brayton cycle (HBC) and transcritical CO_2_ (TCO_2_) for waste heat recovery is being studied for carbon‐free generation. The performance of the proposed SPT based combined cycle (SPT‐HBC‐TCO_2_ cycle) is compared with SPT based basic cycle (SPT‐HBC) based on exergoeconomic and thermodynamic analyses. It is concluded that the SPT‐based combined cycle (SPT‐HBC‐TCO_2_ cycle) produces a thermal efficiency of 32.39% and exergy efficiency of 34.68% with an electricity cost of 1.613 UScent kWh^−1^. The exergy and thermal efficiency of the SPT‐based combined cycle are enhanced by 13.18% and 13.21% respectively, while electricity cost is reduced by around 2% as compared to the SPT‐based basic cycle (SPT‐HBC) configuration at base conditions. A notable finding is that, despite the additional expenditures related to the bottoming cycle, the cost of electricity is lesser for the proposed combined cycle. Additionally, a comparison with the related prior published research exhibits that the performance of the current novel system is superior to that of the systems based on steam rankine cycle and supercritical CO_2_ cycles.

## Graphical abstract

A novel combined power cycle for solar power tower (SPT) system consisting of helium Brayton cycle (HBC) and transcritical CO_2_ (TCO_2_) for waste heat recovery is studied for carbon‐free generation. The performance of the proposed SPT based combined cycle (SPT‐HBC‐TCO_2_ cycle) is compared with SPT based basic cycle (SPT‐HBC) based on exergoeconomic and thermodynamic analyses.

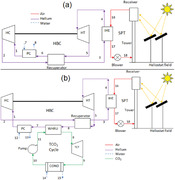

## 1 Introduction

Applications of renewable energy must be developed due to the increasing adverse impact of fossil fuels on the atmosphere.^[^ ^1^, ^2^ ^]^ In this sense, solar energy, the largest source of non‐conventional energy, could be viewed as a trustworthy alternative to the conventional energy sources. The advancement of solar energy consumption is essential for addressing the problems caused by global warming and fossil fuels as well as the rising electricity demand. To generate electricity by utilizing solar energy, two technologies, Concentrating Solar Power (CSP) and Photovoltaic (PV) plants are employed. From the solar radiation, the thermal energy is utilized in CSP‐based systems to power the thermodynamic cycle.^[^ ^2^, ^3^, ^4^ ^]^ Temperatures between 150 and 1500 °C are obtained by CSP using specific mirror arrangements or reflectors in CSP systems.^[^ ^5^ ^]^

Recent research has revealed that among the numerous CSP technology types, SPT based plants are evolving more rapidly than other CSP technologies and garnering more attention.^[^ ^6^ ^]^ Many plane mirrors (heliostats) are installed in these plants to reflect the radiated heat from the sun into a central receiver, where the heat transfer fluid (HTF) receives heat energy. In comparison to parabolic trough collectors, these systems achieve extraordinarily high temperatures (≈1500 °C) due to their high concentration ratios. These systems can operate in a hybrid mode by coupling with traditional fossil fuel plants like coal‐fired Rankine cycles because of their high source temperature resulting in high power generation efficiency.^[^ ^7^ ^]^ Even though solar power towers can be used in the operation of thermodynamic cycles operating at higher temperature range (800−1200 °C) there is no substantial research that addresses this gap of converting high‐temperature energy into electricity using solar power towers.

The analysis of many operational features of solar STP plants has received much research attention since the last decade. The assessment of different receiver types, heliostat field configuration, HTF, and power production systems are the primary components of these research efforts. Studies are being done in this area to propose, develop, and evaluate various power‐producing units in order to achieve higher efficiency.^[^ ^8^ ^]^ Depending on the type of heat source and temperature, the closed Brayton cycles and conventional Rankine cycles can be employed in SPT Plants to convert heat into electricity. In the recent years, supercritical carbon dioxide cycle drew the attention of many researchers due to its advantageous characteristics like compactness and effective performance.^[^ ^9^, ^10^ ^]^ Li et al.^[^ ^11^ ^]^ provided a thorough categorization of sCO_2_ systems and their benefits for application in nuclear and solar generating facilities. In a study conducted by Gkountas et al.,^[^ ^12^ ^]^ for enhancement of heat transfer using Al_2_O_3_ nanofluid concluded that there is a reduction in the length of the heat exchanger by 0.9% and a decrease in a pressure drop by 14% due to application of printed circuit heat exchanger for sCO_2_ systems. The ideal material for heat exchangers is stainless steel up to an operating temperature of 650 °C, whereas nickel‐based alloys are suggested for usage at higher temperatures, according to a detailed evaluation of heat exchanger features employed in helium and sCO_2_ Brayton cycles by Chai and Tassuo.^[^ ^13^ ^]^ The authors Al‐Sulaiman and Atif^[^ ^14^ ^]^ examined five different sCO_2_ cycle designs and modelled the SPT system and concluded that the recompression cycle had the best efficiency (52%) of all the cycles under consideration. The same authors also optimized the arrangement of heliostats in heliostatic field for SPT application.^[^ ^15^ ^]^ Osorio^[^ ^16^ ^]^ examined another sCO_2_ system for the SPT plant. They discovered that 21% of maximal efficiency was attained under diverse seasonal circumstances. Linares et al.’s^[^ ^17^ ^]^ innovative sCO_2_ cycle is much more compact and requires less capital due to the availability of thermal energy at low pressure. At an expense of 8742$ kWe^−1^ for the intercooled reheat system, they evaluated various cycle topologies and concluded that the highest cycle efficiency (52.5%) was achieved with the reheat and intercooling recompression cycle. Wang et al.^[^ ^18^ ^]^ examined five innovative sCO_2_ cycle configurations, involving a basic recuperation cycle, partially cooled, pre‐compression, and intercooled and recompression, for particular work and performed multi‐objective optimization. By using exergoeconomic optimization, Ma et al.,^[^ ^19^ ^]^ discovered that the exergy cost per unit may be reduced by 8.94% in an SPT plant that was coupled to the main compression intercooling recompression sCO_2_ Brayton cycle compared to the basic Brayton cycle. Zahedi et al.^[^ ^20^ ^]^ proposed a second SPT plant with a multi‐generation system centered on sCO_2_ and Rankine power stations. Also, an exergoeconomic study was conducted on an SPT plant with a sCO_2_ Brayton cycle coupled to a thermo‐chemical storage unit by Guelpa and Verda.^[^ ^21^ ^]^ They concentrated on exergoeconomic evaluation at the component level and identified the factors that primarily influence the price per unit of electricity. To generate freshwater and power in an SPT plant, Gang et al.^[^ ^22^ ^]^ carried out a cost analysis of the desalination system combined with sCO_2_ cycle and obtained levelized costs of 1.15 $ t^−1^ and 0.059 $ kWh^−1^ for freshwater and energy, respectively. For an SPT plant with a 10 MW capacity, Chen et al.^[^ ^23^ ^]^ examined off‐design and design circumstances for different sCO_2_ designs. They demonstrated how choosing the ideal configuration requires taking into account off‐design operation situations. Liang et al.^[^ ^24^ ^]^ carried out environmental and economic assessments and multi‐objective optimization for a recompression sCO_2_ (R‐sCO_2_) cycle for SPT plants, and they concluded the 114.8 $MWh^−1^ was the lowest levelized cost of electricity (LCOE).

In the field of hybrid heat source systems, Stand et al.^[^ ^25^ ^]^ proposed a sCO_2_ cycle integrated with the ORC for a hybrid SPT plant. Their study reported the exergy efficiency of the combined cycle was found to be 26.6%. Four sCO_2_ cycle configurations for SPT were subjected to economic and technique assessment and load‐matching calculations by Yang et al.,^[^ ^26^ ^]^ in conjunction with wind and PV hybrid systems. They discovered that the lowest LCOE was associated with a partial‐cooling configuration with 0.217 UScent kWh^−1^. In a different study, they assessed the recuperative sCO_2_ layout's off‐design performance and found that the solar to electrical efficiency of this system was found as 17.8% (in summer) and 17.1% (in winter).^[^ ^27^ ^]^ Jingze et al.^[^ ^28^ ^]^ developed design requirements of the plant by following the performance annually by taking solar radiation changes and the demand of various power situations into account. Two unique combined gas turbine/sCO_2_ systems, using the sCO_2_ cycle as the gas turbine's bottoming cycle, were proposed by Mohammadi et al.^[^ ^29^ ^]^ According to their findings, the Rankine cycle produces higher LCOE than that of the sCO_2_ cycle. A novel optimization approach for a small‐scale SPT combined with a sCO_2_ cycle was provided by Milani et al.^[^ ^30^ ^]^ Their model predicted a 52.7% thermal efficiency for an SPT with a 10 MWe capacity and an 80% cooling water cost savings. A unique cascade method for SPT plants was developed by Yang et al.^[^ ^31^ ^]^ They incorporate the steam Rankine cycle (SRC) with the simple recuperated sCO_2_ cycle. They found that the unique cascade system generates 9.5% more power when compared to the basic sCO_2_ cycle. A combination of sCO_2_ Brayton/ORC for the SPT plant was proposed by Liang et al.,^[^ ^32^ ^]^ in which for the waste heat recovery ORC was used in the two different sCO_2_ system configurations (intercooled and recompression). The combined cycle in the former setup increases output power by 4.4%, while it increases by 3.5% in the latter. Zhu and Wang^[^ ^33^ ^]^ carried out a thermal examination of different direct‐heated sCO_2_ configurations for SPT and its performance was compared, demonstrating that the intercooled arrangement produces the layout's maximum overall efficiency. For the SPT plant, Javanshir et al.^[^ ^34^ ^]^ evaluated the thermodynamics of basic and regenerative Brayton cycles while taking into account a variety of working fluids, including Ne, Kr, He, N_2_ Air, and CO_2_. They discovered that, given the particular output work, helium is the optimal working fluid. Sachdeva and Singh^[^ ^35^ ^]^ designed and analyzed a triple coupled cycle for SPT plants that includes SRC, an air Brayton cycle, and an ORC, and a maximum efficiency of 33.15% was achieved. Guo et al.^[^ ^36^ ^]^ studied CO_2‐_based binary mixtures for solar tower systems. They conducted a comparative study for four different cycle layouts. The binary mixture of CO_2_/Xenon and CO_2_/butane was compared with the sCO_2_ fluid. Their study concluded that the intercooling configuration with CO_2_/Xenon shows the best performance with an exergy efficiency of around 1.32% higher as compared to the sCO_2_ intercooling cycle. By using air as the HTF in a recompression sCO_2_ cycle for an SPT plant, Trevisan et al.^[^ ^37^ ^]^ were able to bypass the temperature restrictions developed by molten salts. Their findings demonstrated that the receiver efficacy is the most important factor and that an LCOE of 100 $ MWh^−1^ is feasible under normal operating conditions. Niu et al.^[^ ^38^ ^]^ examined the use of binary mixtures based on CO_2_ in an SPT in which the power generation unit supercritical Brayton cycle (SBC) was used. They discovered that, for high ambient temperatures, the CO_2_‐propane mixture performs better than other mixtures. Khatoon and Kim^[^ ^39^ ^]^ created a MATLAB program for the construction and simulation of an sCO_2_ power system integrated with SPT. They estimated the net output power for the regenerative and recompression cycles to be 37.17 and 39.04 MW, respectively. Wan et al.^[^ ^40^ ^]^ analyze the off‐design operation of an SPT integrated into the R‐sCO_2_ cycle using a regression learner for tower performance prediction. By using their suggested off‐design control technique, they discovered a 1.02% power gain. Lu et al.^[^ ^41^ ^]^ examined the behavior of the sCO_2_ cycle under off‐design conditions for solar applications. They compared the regenerative and recompression models and concluded that the recompression configuration with a specific bypass fraction showed superior performance under various off‐design circumstances. A solar‐powered sCO_2_ cycle (pre‐compression configuration) connected to an ORC was thermo‐economically evaluated by Khan et al.^[^ ^42^ ^]^ Also, they employed low GWP fluids in the ORC. They discovered that R1336mzz had the lowest specific investment cost, which was determined to be 2234 € kWe^−1^. In order to find the best mole percentage of SF6 in the combination, Bai et al.^[^ ^43^ ^]^ examined the performance of SBCs powered by solar energy in which a mixture of CO_2_‐SF6 was considered a working fluid. Ma et al.^[^ ^44^ ^]^ examined the performance of many other CO_2_‐based combinations in the sCO_2_ Brayton cycle. They concluded that the mixture of CO_2_‐Xe had the best thermodynamically performing system. According to Liu et al.,^[^ ^45^ ^]^ the maximum average daily efficiency for an SPT based on the sCO_2_ Brayton cycle is calculated to be 26.26%. Kademi et al.,^[^ ^46^ ^]^ analyzed the multigenerational system using the multi‐effect distillation unit, ORC, and sCO_2_ Brayton cycle operated by the SPT system. They calculated the optimum exergy efficiency of 61.8% of the combined plant.

Most of the literature cited above has either examined the steam Rankine cycle or the recently developed sCO_2_ cycle for SPT application. The recuperated HBC, which has demonstrated to be one of the most effective and least complicated cycles operating at temperatures over 500 °C, has not been adequately focused in SPT applications, which provides an opportunity to address this research gap. Also, the supplied literature survey demonstrates the volume of studies on the examination of sCO_2_ Brayton cycles for SPT (more than 20 papers in the last 3–4 years) were performed in the field of CSP systems. The application of different gases as the working media in the SBC was examined in a variety of publications,^[^ ^25^, ^34^, ^47^, ^48^, ^49^ ^]^ and it has been demonstrated that helium gas produces better results than CO_2_ as well as other fluids in SBC systems associated with SPTs.

From the above literature review, it is evident that even though HBCs are quite mature in technology their application is restricted due to higher operating temperature and are efficient only when operated at higher temperature due to large back work ratio. Helium has a superior economic benefit as compared to other working fluids for a simple recuperative Brayton configuration.^[^ ^50^ ^]^ The increased heat capacity of helium at higher temperatures results in a reduction of helium mass flow rate and consequently reduces the sizes of components and costs, which is primarily responsible for the improved economic performance of helium as a working fluid. The SPT plants generally operate at higher temperatures and helium has shown better performance as compared to carbon dioxide at these operating temperatures.^[^ ^51^ ^]^

The literature study does not provide much evidence related to the application of the HBC in solar thermal plants. Traditionally, the ORC has been most preferred for low‐temperature applications due to the favorable operating conditions of the various organic fluids. The TCO_2_ cycle provides a better option to recover heat from the high‐temperature heat source over the traditionally used ORC cycles as it has a better temperature‐matching glide in the evaporator over organic fluid. The application of organic fluid leads to pinch‐point temperature problems in the evaporator. In terms of the comparable thermodynamic mean heat rejection temperature, TCO_2_ outperformed ORC.^[^ ^52^ ^]^ Hence, a novel system that comprises of HBC and TCO_2_ cycle has been investigated from the exergoeconomic and thermodynamic point of view in the present study. In the present study, using helium fluid, SBC performance has been significantly improved. At temperatures between 150 and 250 °C, a significant quantity of thermal energy is lost to the atmosphere in SBC systems in order to cool the working fluid at the compressor entrance and reduce compression power consumption. To recover waste heat, as a bottoming cycle to HBC, the TCO_2_ cycle is used. Several works^[^ ^53^, ^54^, ^55^ ^]^ have examined the versatility of the TCO_2_ cycle to recover waste heat.

Regarding the discussion above, the primary aims of the current study are:

a) To propose an efficient and novel SBC system for SPT plants using helium working fluid in which the TCO_2_ cycle is used as the bottoming cycle for extra power generation.
b) Thermodynamic and exergoeconomic analyses, and cost of electricity calculation for both the standalone HBC and the proposed HBC‐TCO_2_ combined cycle.
c) Balancing increased expenses (related to the TCO_2_ cycle) with improved electricity generation.

The following points are some of the significant constraints imposed by the present study:

a) The research method of the current study is theoretical. An experiment is not performed due to funding constraints.
b) Unsteady conditions during the start‐up stage were not taken into account during the system's modeling because it was done under steady‐state operation.
c) In line with related earlier studies, modeling of the heat exchangers are done taking the reasonable effectiveness values.
d) Factors that should be taken into consideration in related future research were not taken into account related to the energy‐environmental evaluation of the proposed system.

## 2 System Description

**Figure 1:**
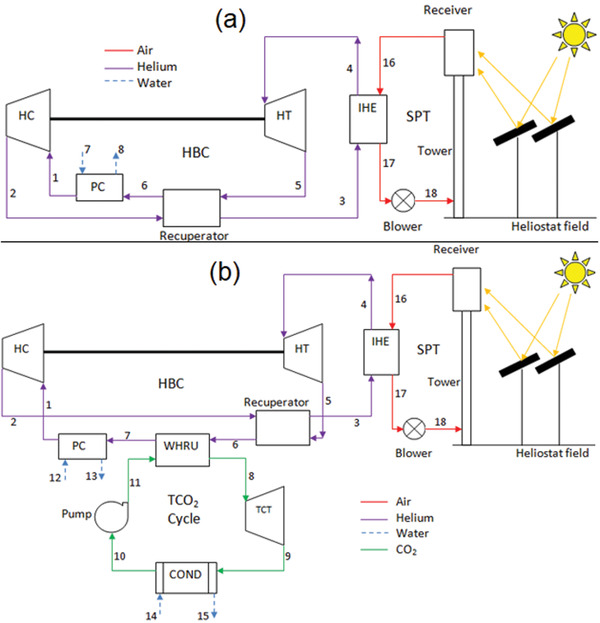
Schematic of diagrams of SPT based a) basic HBC system and b) proposed combined HBC‐TCO_2_ cycle.

**Figure** **1**a shows the systematic diagram of HBC integrated with SPT and Figure 1b represents the combined HBC‐TCO_2_ integrated with SPT to produce additional power. As shown in Figure 1a, there are two subsections of the system, first is the solar subsystem (comprising of blower, receiver, and heliostat field) and the other is the HBC sub‐system (consisting of compressor, turbine, precooler, recuperator, and internal heat exchanger).The HTF is heated in the volumetric receivers to the temperature of ≈1000 °C^[^ ^56^ ^]^ in the solar tower by reflecting the solar intensity from the heliostats. In the present research, an open‐type volumetric air receiver is used as a commercial and standardized receiver, so air acts as an HTF in the receiver. The temperature restriction and receiver design complexity imposed by using molten salt as HTF can be avoided by using air as the HTF.^[^ ^56^ ^]^ The heat energy from the volumetric receivers is transferred by HTF to the HBC through IHE (Process 18‐16). It can be observed from Figure 1a that helium in its supercritical form enters the compressor and its temperature and pressure rise due to compression of fluid (Process 1–2). The helium heats up in two different stages after the exit from the compressor and before entering the turbine. Initially, the helium is heated in the recuperator (Process 2–3) and then it is heated in the IHE by the HTF (Process 3–4) to reach the desired turbine inlet temperature. The high‐temperature, high‐pressure helium is then expanded in the helium turbine to generate power (Process 4–5). After the expansion, the low‐pressure and moderate‐temperature helium passes through the recuperator to exchange heat (Process 5–6). The hot stream is then passed through the precooler to lose its remaining heat and then enters the compressor (Process 6‐1). A significant of heat is lost to the environment in cooling the helium. Figure 1b depicts the combined HBC‐TCO_2_ system where an additional transcritical carbon dioxide cycle is added to the system shown in Figure 1a. After passing through the recuperator the helium flowing stream has a high temperature of around 220 °C. Referring to Figure 1b, in order to recover the waste heat and improve the overall efficiency of the combined system, a transcritical carbon dioxide cycle is combined with the helium Brayton cycle through a waste heat recovery unit (WHRU). The hot stream after the recuperator passes through the WHRU to provide heat to the TCO_2_ cycle (Process 6–7). The helium is then cooled in the precooler (Process 7‐1). As a result of heat exchange in the WHRU, the CO_2_ gets heated up by gaining heat from the helium (Process 8–11). The high‐temperature, high‐pressure CO_2_ is then expanded in the transcritical turbine to generate power (Process 8–9). The low‐temperature, low‐pressure CO_2_ then loses its remaining heat in the condenser (Process 9–10). The low‐pressure CO_2_ then enters the pump to get pumped to the high pressure (Process 10–11). The high‐pressure CO_2_ then enters the WHRU to gain heat and the process repeats. Since, both the cycles are operating in a closed loop manner, resulting in carbon‐free power generation.

## 3 Thermodynamic and Exergoeconomic Analysis

### 3.1 Assumptions

The examination of the present systems has taken into account the following presumption: 1) All components are operated in a steady state condition.^[^ ^56^ ^]^ 2) As stated in **Table** **1**, pressure loss in the components is anticipated. 3) Kinetic and potential energy were neglected.^[^ ^66^ ^]^ 4) The blower in the SPT system is responsible for a negligible change in thermodynamic properties, therefore thermodynamic properties at the states 17 and 18 are almost the same.^[^ ^56^ ^]^ 5) Isentropic efficiency for the pump, turbine, and compressor, are assumed and shown in Table 1.

**Table 1:** Input data of the proposed system.

Parameter	Value
Receiver efficiency (η_rec_)	0.9^[^ ^66^ ^]^
Receiver aperture area (A_rec_)	68.1 m^2[^ ^57^ ^]^
The reflective area of each heliostat (A_hel_)	9.45 × 12.84 m^2[^ ^57^ ^]^
Direct normal irradiation (DNI)	850 W m^−2[^ ^56^, ^57^ ^]^
Pressure at the inlet of HC (P_1_)	2500 kPa^[^ ^56^ ^]^
Number of heliostats (N_hel_)	500^[^ ^57^ ^]^
Heliostat field efficiency (η_field_)	0.75^[^ ^66^ ^]^
The temperature at the inlet of HT(T_4_)	850 °C^[^ ^56^, ^57^ ^]^
HC isentropic efficiency (η_HC_)	0.88^[^ ^63^ ^]^
Heat exchanger effectiveness(ε))	0.9^[^ ^56^, ^63^ ^]^
Apparent Sun temperature (T_Sun_)	4500 K^[^ ^56^ ^]^
HT isentropic efficiency (η_HT_)	0.9^[^ ^63^ ^]^
Pinch point difference in condenser	5 °C^[^ ^70^ ^]^
Isentropic efficiency of a pump (η_Pump_)	0.8^[^ ^70^ ^]^
The temperature at the inlet of TCO_2_ turbine (T_8_)	180 °C^[^ ^56^ ^]^
Compressor pressure ratio (CPR)	2.5
Isentropic efficiency of TCO_2_ turbine (η_TCT_)	0.85^[^ ^70^ ^]^
Pinch point difference in WHRU	10 °C^[^ ^56^ ^]^
Atmospheric temperature (T_0_)	25 °C^[^ ^56^, ^70^ ^]^
Atmospheric pressure (P_0_)	101.3 kPa^[^ ^56^, ^70^ ^]^
Pressure loss in IHE	2%^[^ ^56^ ^]^
Pressure loss in recuperator/WHRU	1%^[^ ^56^ ^]^

### 3.2 Thermodynamic Evaluation

For thermodynamic investigation, the overall combined system is subdivided into two systems namely solar sub‐system and thermodynamic power cycle. A thermodynamic model has been developed for each component, and simulation is performed by the computational technique developed in the Engineering Equation Solver (EES) software. The energy and exergy balancing equations are written following a control volume approach and steady‐state process:^[^ ^34^, ^35^, ^36^, ^37^, ^38^, ^39^, ^40^ ^]^

(1) $${\dot{Q}}_{CV}-{\dot{W}}_{CV}+\sum \left({\dot{m}}_{i}h_{i}\right)-\sum \left({\dot{m}}_{e}h_{e}\right)=0$$

(2) $$\overset{\cdot }{EXD}={\overset{\cdot }{EX}}_{\mathrm{in}}-{\overset{\cdot }{EX}}_{\mathrm{out}}$$

where ${\overset{\cdot }{EX}}_{\mathrm{in}}$ and ${\overset{\cdot }{EX}}_{\mathrm{out}}$stands for the rate of total exergy that enters and leaves the control volume, respectively, while $\overset{\cdot }{EXD}$ represents the amount of exergy destruction rate within the component. Neglecting the changes in kinetic and potential energy and because the chemical exergy is nullified in the exergy balance equations (since no chemical reactions are occurring in the system under consideration) the flow exergy rate of the fluid stream is equal to the physical exergy and can be presented as;^[^ ^34^, ^35^, ^36^, ^37^, ^38^, ^39^, ^40^ ^]^

(3) $${\overset{\cdot }{EX}}_{i}=\overset{\cdot }{m}\cdot \left[\left(h_{i}-h_{0}\right)-T_{0}\left(s_{i}-s_{0}\right)\right]$$

where “*i*” referes to specific state and ${\overset{\cdot }{EX}}_{i}$ is the *i* ^th^ state physical exergy.

The solar sub‐system comprises two parts, i.e., the heliostat field and the solar receiver. The heliostat field consists of several heliostats with a total aperture area of *A*_hel_ that concentrates DNI on a tower's central receiver using a point‐focused technology. The amount of solar thermal energy reaching the central receiver depends on the intensity of solar radiation and the efficiency of the heliostat field. The solar radiation variation depends on factors like geographical location and time of the day whereas the efficiency of a heliostat field depends on the field layout and number of heliostats. The receiver absorbs the remaining total DNI as useable heat whereas a portion of the total DNI emitted on the heliostats is lost in the field. Consequently, the receiver's thermal response to the heliostat field can be represented as;^[^ ^52^, ^53^ ^]^

(4) $${\dot{Q}}_{\mathrm{rec},\;\mathrm{in}}=\eta_{\mathrm{field}}\cdot {\dot{Q}}_{\mathrm{Sun}}=\eta_{\mathrm{field}}\times DNI\times A_{\mathrm{hel}}\times N_{\mathrm{hel}}$$

where optical heliostat field efficiency is denoted by η_field_ and can be expressed as;^[^ ^52^, ^53^ ^]^

(5) $$\eta_{\mathrm{field}}=\eta_{\cos}\cdot \eta_{s\&b}\cdot \eta_{int}\cdot \eta_{\mathrm{att}}\cdot \eta_{\mathrm{ref}}$$

where $\eta_{s\&b}$, η_cos_, η_int_, η_att_, and η_ref_ represents the efficiencies of theshading and blocking, cosine effect, interception efficiency, atmospheric attenuation, and heliostats reflectivity, respectively. It is important to highlight that the computation of these parameters is beyond the scope of the current article and the practical values of an existing solar plant are considered for the above‐mentioned parameters in the present paper. These values have been referred to in literature.^[^ ^56^, ^57^, ^62^ ^]^

The ${\dot{Q}}_{\mathrm{rec},\;\mathrm{in}}$ is the total amount of heat received by the receiver and ${\dot{Q}}_{\mathrm{rec},\;\mathrm{loss}\;}$ is the amount of heat energy that is lost to the environment due to conduction, convection, and radiation. The net energy remaining ${\dot{Q}}_{\mathrm{rec},\;\mathrm{net}}$ after the loss in the receiver is then transferred to the HTF. For the central receiver, the energy efficiency and energy balance can be represented as^[^ ^56^ ^]^

(6) $$\eta_{\mathrm{rec}}=\frac{{\dot{Q}}_{\mathrm{rec},\;\mathrm{net}}}{{\dot{Q}}_{\mathrm{rec},\;\mathrm{in}}}$$

(7) $${\dot{Q}}_{\mathrm{rec},\;\mathrm{in}}={\dot{Q}}_{\mathrm{rec},\;\mathrm{net}}+{\dot{Q}}_{\mathrm{rec},\;\mathrm{loss}}={\dot{m}}_{\mathrm{air}}\left(h_{16}-h_{17}\right)+{\dot{Q}}_{\mathrm{rec},\;\mathrm{loss}}$$

The relations that were used to analyze the system component using thermodynamic analysis is are listed in **Table** **2**. These equations are all incorporated into the simulation code created with EES, along with the input data listed in Table 1. The program extracts thermodynamic properties in order to determine each of the unknown variables, such as state point thermodynamic properties, work and heat interactions, and the rate of exergy at each state. The overall thermal and exergy efficiency for the present solar thermal power plant has been expressed as the ratio of net power output to the input heat or exergy available from solar irradiation on the heliostat field:^[^ ^2^, ^55^, ^56^, ^57^ ^]^

(8) $$\eta_{\mathrm{th},\;\mathrm{overall}}=\frac{{\dot{W}}_{\mathrm{net}}}{{\dot{Q}}_{\mathrm{Sun}}}$$

(9) $$\eta_{\mathrm{ex},\;\mathrm{overall}}=\frac{{\dot{W}}_{\mathrm{net}}}{{\dot{Q}}_{\mathrm{Sun}}\cdot \left(1-\frac{T_{0}}{T_{\mathrm{ref},\;\mathrm{Sun}}}\right)}$$

where *T*_ref,Sun_ is the apparent sun temperature (4500 K).^[^ ^56^ ^]^ For exergy analysis, it was considered as the equivalent temperature of the heat source.^[^ ^52^, ^53^ ^]^ ${\dot{W}}_{\mathrm{net}}$ for the overall power plant (SPT‐HBC‐TCO_2_ cycle) can be evaluated as^[^ ^42^, ^56^ ^]^:

(10) $${\dot{W}}_{\mathrm{net}}={\dot{W}}_{\mathrm{HT}}-{\dot{W}}_{\mathrm{HC}}+{\dot{W}}_{\mathrm{TCT}}-{\dot{W}}_{\mathrm{pump}}$$

**Table 2:** Energy and exergy analyses equations of each component.

Component	Energy equations	Exergy equations
Heliostat field	${\dot{Q}}_{rec,\;in}=\eta_{field}\cdot \;DNI\;\cdot A_{hel}\cdot N_{hel}$	${\dot{Q}}_{Sun}\cdot \left(1-\;\frac{T_{0}}{T_{ref,Sun}}\right)={\dot{Q}}_{rec,\;in}\cdot \left(1-\;\frac{T_{0}}{T_{ref,hel}}\right)+{\overset{\cdot }{ED}}_{hel}$
Receiver	${\dot{Q}}_{rec,\;in}={\dot{m}}_{air}\left(h_{12}-h_{17}\right)+{\dot{Q}}_{rec,\;loss}$	${\overset{\cdot }{EX}}_{17}+{\dot{Q}}_{rec,\;in}\cdot \left(1-\;\frac{T_{0}}{T_{ref,hel}}\right)={\overset{\cdot }{EX}}_{16}+{\dot{Q}}_{rec,\;loss}\cdot \left(1-\;\frac{T_{0}}{T_{rec}}\right){\overset{\cdot }{+ED}}_{rec}$
IHE	${\dot{Q}}_{IHE}={\dot{m}}_{air}\cdot \left(h_{16}-h_{17}\right)+{\dot{m}}_{He}\cdot \left(h_{4}-h_{3}\right)$	${\overset{\cdot }{EX}}_{16}-{\overset{\cdot }{EX}}_{17}=\;{\overset{\cdot }{EX}}_{4}-{\overset{\cdot }{EX}}_{3}+{\overset{\cdot }{ED}}_{IHE}$
Helium turbine (HT)	${\dot{W}}_{HT}={\dot{m}}_{He}\cdot \left(h_{4}-h_{5}\right)$ $\eta_{HT}=\frac{\left(h_{4}-h_{5}\right)}{\left(h_{4}-h_{5s}\right)}$	${\overset{\cdot }{EX}}_{4}=\;{\overset{\cdot }{EX}}_{5}+{\dot{W}}_{HT}+{\overset{\cdot }{ED}}_{HT}$
Helium compressor (HC)	${\dot{W}}_{HC}={\dot{m}}_{He}\cdot \left(h_{2}-h_{1}\right)$ $\eta_{HC}=\frac{\left(h_{2s}-h_{1}\right)}{\left(h_{2}-h_{1}\right)}$	${\overset{\cdot }{EX}}_{1}=\;{\overset{\cdot }{EX}}_{2}-{\dot{W}}_{HC}+{\overset{\cdot }{ED}}_{HC}$
Recuperator	(*h*_3_ − *h*_2_) = (*h*_5_ − *h*_6_) $\varepsilon_{recuperator}=\frac{\left(T_{3}-T_{2}\right)}{\left(T_{5}-T_{2}\right)}$	${\overset{\cdot }{EX}}_{5}-{\overset{\cdot }{EX}}_{6}=\;{\overset{\cdot }{EX}}_{3}-{\overset{\cdot }{EX}}_{2}+{\overset{\cdot }{ED}}_{recuperator}$
WHRU	${\dot{m}}_{He}\cdot \left(h_{6}-h_{7}\right)={\dot{m}}_{CO2}\cdot \left(h_{8}-h_{11}\right)$	${\overset{\cdot }{EX}}_{6}-{\overset{\cdot }{EX}}_{7}=\;{\overset{\cdot }{EX}}_{8}-{\overset{\cdot }{EX}}_{11}+{\overset{\cdot }{ED}}_{WHRU}$
Precooler	${\dot{m}}_{air}\cdot \left(h_{7}-h_{1}\right)={\dot{m}}_{water}\cdot \left(h_{13}-h_{12}\right)$	${\overset{\cdot }{EX}}_{7}-{\overset{\cdot }{EX}}_{1}=\;{\overset{\cdot }{EX}}_{13}-{\overset{\cdot }{EX}}_{12}+{\overset{\cdot }{ED}}_{Precooler}$
Condenser (COND)	${\dot{m}}_{CO2}\cdot \left(h_{9}-h_{10}\right)={\dot{m}}_{water}\cdot \left(h_{15}-h_{14}\right)$	${\overset{\cdot }{EX}}_{9}-{\overset{\cdot }{EX}}_{10}=\;{\overset{\cdot }{EX}}_{15}-{\overset{\cdot }{EX}}_{14}+{\overset{\cdot }{ED}}_{COND}$
TCO_2_ turbine (TCT)	${\dot{W}}_{TCT}={\dot{m}}_{CO2}\left(h_{8}-h_{9}\right)$ $\eta_{TCT}=\frac{\left(h_{8}-h_{9}\right)}{\left(h_{8}-h_{9s}\right)}$	${\overset{\cdot }{EX}}_{8}=\;{\overset{\cdot }{EX}}_{9}+{\dot{W}}_{TCT}+{\overset{\cdot }{ED}}_{TCT}$
Pump	${\dot{W}}_{Pump}={\dot{m}}_{CO2}\left(h_{11}-h_{10}\right)$ $\eta_{Pump}=\frac{\left(h_{11s}-h_{10}\right)}{\left(h_{11}-h_{10}\right)}$	${\overset{\cdot }{EX}}_{10}=\;{\overset{\cdot }{EX}}_{11}-{\dot{W}}_{Pump}+{\overset{\cdot }{ED}}_{Pump}$

Furthermore, each component or subsystem's exergy efficiency can be expressed using the product $\left({\overset{\cdot }{EX}}_{\mathrm{Product}}\right)$ and fuel exergy $\left({\overset{\cdot }{EX}}_{\mathrm{Fuel}}\right)$ concepts as follows:

(11) $$\eta_{\mathrm{ex}}=\frac{{\overset{\cdot }{EX}}_{\mathrm{Product}}}{{\overset{\cdot }{EX}}_{\mathrm{Fuel}}}$$

The thermodynamic combined cycle (HBC‐TCO_2_ cycle) is the power generation unit (PGU) that converts thermal energy consumed in IHE to mechanical energy. Its efficiencies can be defined by the fuel and product exergy definition:

(12) $$\eta_{\mathrm{th},\;\mathrm{PGU}}=\frac{{\dot{W}}_{\mathrm{net}}}{{\dot{Q}}_{\mathrm{IHE}}}$$

(13) $$\eta_{\mathrm{ex},\;\mathrm{PGU}}=\frac{{\dot{W}}_{\mathrm{net}}}{\left({\overset{\cdot }{EX}}_{16}-{\overset{\cdot }{EX}}_{17}\right)}$$

where $\left({\overset{\cdot }{EX}}_{16}-{\overset{\cdot }{EX}}_{17}\right)$ denotes available exergy input to the PGU.

The exergy destruction ratio (*y*_D_) is another energetic performance parameter that explains the weak point of the system, which can be expressed as^[^ ^59^ ^]^:

(14) $$y_{D}=\frac{{\overset{\cdot }{EXD}}_{j}}{{\overset{\cdot }{EX}}_{\mathrm{in},\;j}}$$

where ${\overset{\cdot }{EX}}_{\mathrm{in},\;j}$ and ${\overset{\cdot }{EXD}}_{j}$ are exergy rate input and destruction of the *j* ^th^ component, respectively.

The overall heat transfer coefficient's approximate values are used for calculating the heat exchanger area. For the recuperator, a value of 3.0 kW m^−2^ °C^−1^ is taken into account. A value of 1.6 kW m^−2^ °C^−1^ is considered for the precooler and WHRU. Moreover, the estimated value of 2.0 kW m^−2^ °C^−1^ is employed for the condenser.^[^ ^58^ ^]^

### 3.3 Exergoeconomic Analysis

Thermodynamic analysis for any system focuses only on the energy conversion which is expressed by the first law and the amount of available energy that is defined by the second law. The economic considerations while designing any system lead to less complex, highly efficient, and cost‐effective systems. Exergy analysis with the economic associated at the level of a system component is called exergoeconomics analysis. The main aim of an exergoeconomic study is to determine the system's overall cost rate by illuminating the cost formation process. Exergoeconomics has been approached from a variety of angles, including the specific exergy costing (SPECO),^[^ ^54^, ^55^ ^]^ the average cost approach,^[^ ^61^ ^]^ and the exergy cost theory.^[^ ^60^ ^]^ The SPECO method is used in this research because of its simple, uncomplicated design and effective calculation using a matrix formulation. It is important to derive the combined systems' cost balance model to determine the cost per kWh. In an exergoeconomic approach, it is crucial to first determine the fuel and product of each component. The fuel and product for the two systems that are suggested in this work are presented in **Table** **3**.

**Table 3:** Product and fuel definition of the proposed SPT‐based combined (HBC‐TCO_2_) cycle.

Component	Fuel [kW]	Product [kW]
Heliostat	${\overset{\cdot }{EX}}_{Sun}$	${\overset{\cdot }{EX}}_{Heliostat}$
Receiver	${\overset{\cdot }{EX}}_{Heliostat}$	${\overset{\cdot }{EX}}_{16}-{\overset{\cdot }{EX}}_{17}$
IHE	${\overset{\cdot }{EX}}_{16}-{\overset{\cdot }{EX}}_{17}$	${\overset{\cdot }{EX}}_{4}-{\overset{\cdot }{EX}}_{3}$
HT	${\overset{\cdot }{EX}}_{4}-{\overset{\cdot }{EX}}_{5}$	${\dot{W}}_{HT}$
Recuperator	${\overset{\cdot }{EX}}_{5}-{\overset{\cdot }{EX}}_{6}$	${\overset{\cdot }{EX}}_{3}-{\overset{\cdot }{EX}}_{2}$
WHRU	${\overset{\cdot }{EX}}_{6}-{\overset{\cdot }{EX}}_{7}$	${\overset{\cdot }{EX}}_{8}-{\overset{\cdot }{EX}}_{11}$
Precooler	${\overset{\cdot }{EX}}_{7}-{\overset{\cdot }{EX}}_{1}$	${\overset{\cdot }{EX}}_{13}-{\overset{\cdot }{EX}}_{12}$
HC	${\dot{W}}_{HC}$	${\overset{\cdot }{EX}}_{2}-{\overset{\cdot }{EX}}_{1}$
TCT	${\overset{\cdot }{EX}}_{8}-{\overset{\cdot }{EX}}_{9}$	${\dot{W}}_{HC}$
Condenser	${\overset{\cdot }{EX}}_{9}-{\overset{\cdot }{EX}}_{10}$	${\overset{\cdot }{EX}}_{15}-{\overset{\cdot }{EX}}_{14}$
Pump	${\dot{W}}_{Pump}$	${\overset{\cdot }{EX}}_{11}-{\overset{\cdot }{EX}}_{10}$

For each system component and cost balance function is expressed as^[^ ^54^, ^55^ ^]^:

(15) $$\sum {\dot{C}}_{in,\;k}+{\dot{C}}_{q,\;k}+{\dot{Z}}_{j}=\sum {\dot{C}}_{out,\;k}+{\dot{C}}_{w,\;k}$$

where the terms ${\dot{C}}_{q,\;k}$ and ${\dot{C}}_{w\;k\;}$ are the cost rates at state point k associated with input thermal energy and output power to and from the component, respectively and ${\dot{Z}}_{j}$ is the investment cost rate of the *j* ^th^ component.

The exergy unit cost for each component associated with the work, heat, and flow rate from the component are illustrated below^[^ ^59^ ^]^:

(16) $$\begin{array}{c} {\dot{C}}_{in,k}=c_{k}\cdot {\overset{\cdot }{EX}}_{k}\\ {\dot{C}}_{q,k}=c_{q}\cdot {\overset{\cdot }{EX}}_{k}\\ {\dot{C}}_{w,k}=c_{w}\cdot \dot{W} \end{array}$$

where *c_k_*, *c_w_*, and *c_q_* are the average costs per unit of exergy.

The cost rate for component *j* is given by:^[^ ^62^ ^]^

(17) $${\dot{Z}}_{j}=\frac{Z_{j}\cdot CRF\cdot \varphi }{3600\cdot N}$$

where *Z_j_* denotes the component's capital cost, CRF denotes the capital recovery factor, φ denotes the maintenance factor (assumed 1.06), and N denotes the system's operating hours annually, which are 7446 h.^[^ ^71^ ^]^

CRF is defined as^[^ ^54^, ^55^ ^]^:

(18) $$\mathrm{CRF}=\frac{i\cdot {\left(1+i\right)}^{n}}{{\left(1+i\right)}^{n}-1}$$

where *i* is the rate of interest which is 12%,^[^ ^63^ ^]^ and “n” stands for the system's life, which is equal to 20 years.^[^ ^63^ ^]^ In **Table** **4**, the capital cost of various components is also displayed. The cost rate balance functions and auxiliary equations for the proposed entire plant are shown in **Table** **5**, where, fuel and product definition are used to establish the auxiliary equations. It is possible to calculate the rate of cost and per unit cost of exergy for each state point by solving the equations in Tables 4 and 5, respectively.

**Table 4:** Investment model of each component of the proposed combined cycle.

Component	Investment model
Heliostat	*Z_hel_* = 126 · *A_hel_* · *N_hel_* ^[^ ^63^ ^]^
Receiver	*Z_rec_* = *A_rec_* · (79 · *T*_16_ − 42000)^[^ ^63^ ^]^
IHE	$Z_{IHE}=12000\cdot {\left(\frac{A_{IHE}}{100}\right)}^{0.6}$ ^[^ ^63^ ^]^
HT	$Z_{HT}=\frac{479.3\cdot {\dot{m}}_{He}}{0.93-\eta_{HT}}\cdot \ln\left(\frac{P_{4}}{P_{5}}\right)\cdot (1+\exp\left(0.036\cdot T_{4}-54.4\right)$ ^[^ ^63^ ^]^
Recuperator	*Z_Recuperator_* = 2681 · (*A_Recuperator_*)^0.59[^ ^63^ ^]^
WHRU	*Z_WHRU_* = 2681 · (*A_WHRU_*)^0.59[^ ^70^ ^]^
Precooler	*Z_precooler_* = 2143 · (*A_precooler_*)^0.514[^ ^63^ ^]^
HC	$Z_{HC}=\frac{71.1\cdot {\dot{m}}_{He}}{0.91-\eta_{HC}}\cdot \left(\frac{P_{2}}{P_{1}}\right)\cdot \ln\left(\frac{P_{2}}{P_{1}}\right)$ ^[^ ^63^ ^]^
TCT	$Z_{TCT}=4405\cdot {\left({\dot{W}}_{TCT}\right)}^{0.7}$ ^[^ ^70^ ^]^
Condenser	*Z_condenser_* = 2143 · (*A_condenser_*)^0.514^^[^ ^70^ ^]^
Pump	$Z_{Pump}=1120\cdot {\left({\dot{W}}_{pump}\right)}^{0.8}$ ^[^ ^70^ ^]^

**Table 5:** Each component cost rate and auxiliary equations.

Component	Cost rate equations	Auxiliary equations
Heliostat	${\dot{C}}_{Sun}+{\dot{Z}}_{hel}={\dot{C}}_{Q,rec}$	${\dot{C}}_{Sun}=0$
Receiver	${\dot{C}}_{Q,rec}+{\dot{C}}_{17}+{\dot{Z}}_{hel}={\dot{C}}_{16}$	*c*_16_ = *c*_17_
IHE	${\dot{C}}_{16}+{\dot{C}}_{3}+{\dot{Z}}_{IHE}={\dot{C}}_{4}+{\dot{C}}_{17}$	*c*_3_ = *c*_4_
HT	${\dot{C}}_{4}+{\dot{Z}}_{IHE}={\dot{C}}_{5}+{\dot{C}}_{W,HT}$	*c*_4_ = *c*_5_
Recuperator	${\dot{C}}_{5}+{\dot{C}}_{2}+{\dot{Z}}_{recuperator}={\dot{C}}_{3}+{\dot{C}}_{6}$	*c*_6_ = *c*_5_
WHRU	${\dot{C}}_{6}+{\dot{C}}_{11}+{\dot{Z}}_{WHRU}={\dot{C}}_{7}+{\dot{C}}_{8}$	*c*_8_ = *c*_11_
Precooler	${\dot{C}}_{7}+{\dot{C}}_{12}+{\dot{Z}}_{recuperator}={\dot{C}}_{13}+{\dot{C}}_{1}$	*c*_1_ = *c*_7_ *c*_12 = 0_
HC	${\dot{C}}_{1}+{\dot{C}}_{W,HC}+{\dot{Z}}_{HC}={\dot{C}}_{2}$	*c*_2_ = *c*_1_
TCT	${\dot{C}}_{8}+{\dot{Z}}_{TCT}={\dot{C}}_{9}+{\dot{C}}_{W,TCT}$	*c*_8_ = *c*_9_
Condenser	${\dot{C}}_{9}+{\dot{C}}_{14}+{\dot{Z}}_{COND}={\dot{C}}_{15}+{\dot{C}}_{10}$	*c*_14 = 0_ *c*_10_ = *c*_9_
Pump	${\dot{C}}_{10}+{\dot{C}}_{W,Pump}+{\dot{Z}}_{Pump}={\dot{C}}_{11}$	‐

A number of crucial characteristics that are based on fuel and product definitions need to be determined to get better information on the exergoeconomic performance of the two systems. Fuel exergy average cost for component *j* is:^[^ ^54^, ^55^ ^]^

(19) $$c_{F,j}=\frac{{\dot{C}}_{F,j}}{{\overset{\cdot }{EX}}_{F,J}}$$

And product exergy average cost for component *j* is:^[^ ^54^, ^55^ ^]^

(20) $$c_{P,j}=\frac{{\dot{C}}_{P,j}}{{\overset{\cdot }{EX}}_{P,j}}$$

The cost rate associated with exergy destruction for component *j* is defined as^[^ ^59^ ^]^:

(21) $${\dot{C}}_{D,j}=c_{F,j}\cdot {\overset{\cdot }{EXD}}_{j}$$

where ${\overset{\cdot }{EXD}}_{j}$ represents the exergy destruction rate for the *j* ^th^ component, represented by^[^ ^59^ ^]^:

(22) $${\overset{\cdot }{EXD}}_{j}={\overset{\cdot }{EXD}}_{F,j}-{\overset{\cdot }{EXD}}_{P,j}$$

The exergoeconomic factor for component *j* shows the investment cost relative to the cost of exergy destruction is expressed as^[^ ^54^, ^55^ ^]^:

(23) $$f_{j}=\frac{{\dot{Z}}_{j}}{{\dot{Z}}_{j}+{\dot{C}}_{D,j}}$$

The cost of electricity per unit can be expressed as^[^ ^54^, ^55^, ^59^ ^]^:

(24) $$C_{W}=\frac{{\dot{Z}}_{j}+{\dot{C}}_{fuel}}{{\dot{W}}_{net}}$$

where ${\dot{C}}_{fuel}\;$is the fuel cost. However, in this system, this refers to the cost of the Sun, which is zero in this study.

### 3.4 Validation

**Figure 2:**
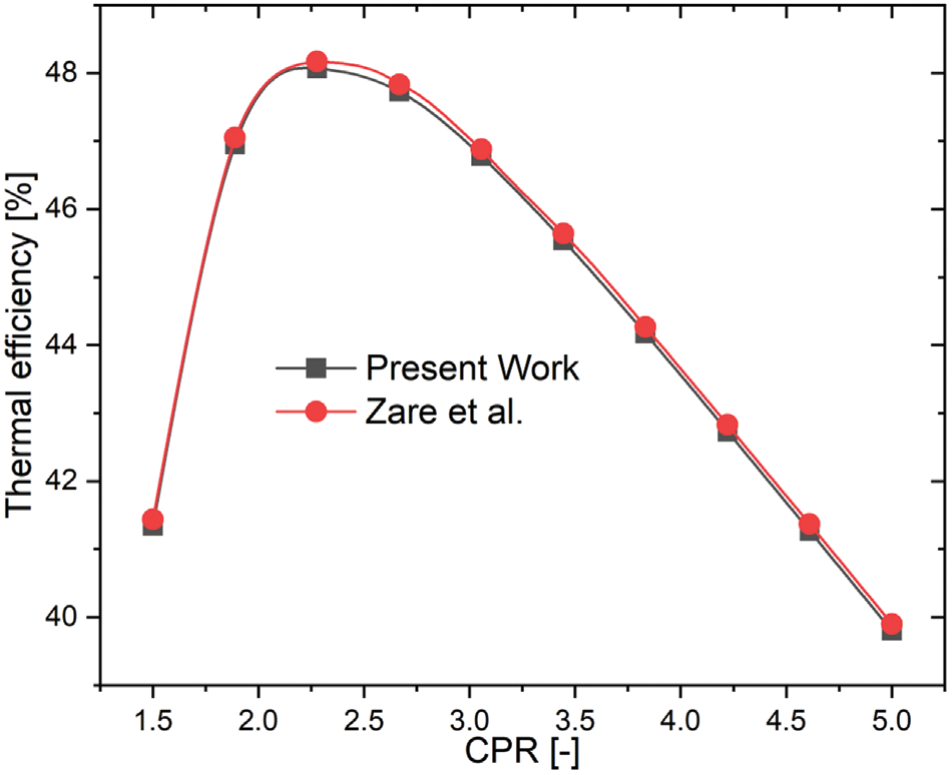
Validation of basic HBC cycle.

To validate the simulation results of both the models (HBC and TCO_2_ cycle) are compared and verified with the previously published literature. **Figure** **2** illustrates the comparisons of efficiency values of the standalone HBC obtained in the current work with those studied by Zare et al.^[^ ^64^ ^]^ Only ≈0.24% of the data differs, which is acceptable. The bottoming TCO_2_ cycle was verified using the results reported in **Table** **6** by Song et al.,^[^ ^65^ ^]^ considering the same input conditions. It can be observed that there is a less than 1% fluctuation in the results, which is likewise acceptable.

**Table 6:** Validation of TCO_2_ cycle.

Parameter^a)^	Present work	Song et al.,^[^ ^65^ ^]^	Deviation (%)
Heat source (kW)	100	100	0
${\dot{W}}_{\mathrm{TCT}}$ (kW)	12.105	12.099	0.04
${\dot{W}}_{\mathrm{Pump}}$ (kW)	3.651	3.648	0.08
η_*th*_ (%)	8.5	8.48	0.23

^a)^ η_TCT_=0.7, η_Pump_=0.8

## 4 Results and Discussion

In the present study, a highly efficient novel combined power generation cycle has been developed to utilize the sun's heat energy using a solar power tower system. The exergy, energy, and exergoeconomic investigation was carried out to examine the feasibility of the proposed system. In this first section comparison with the basic standalone HBC cycle is discussed, followed by energy, exergy and exergoeconomic analyses, and then parametric analysis to find the optimal points. To understand the impact of the present study, a comparative study at the end of this section is presented in Table 12.

### 4.1 Comparison with Basic HBC Cycle

**Figure 3:**
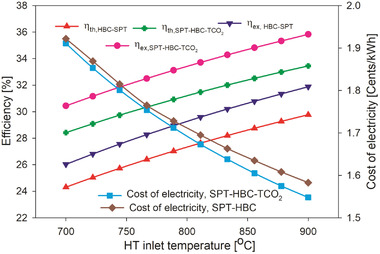
Combined systems (SPT‐HBC‐TCO_2_) performance comparison with SPT‐HBC system.

The proposed SPT‐based combined cycle (SPT‐HBC‐TCO_2_) (overall plant) was compared with the SPT‐based basic HBC system (SPT‐HBC). At the same baseline condition, the proposed SPT‐based combined cycle (SPT‐HBC‐TCO_2_ cycle) was more efficient than SPT‐based basic HBC (SPT‐HBC). The exergy and thermal efficiency of the SPT‐HBC system were found as 30.64% and 28.61% respectively. However, the thermal and exergy efficiency of the overall plant (SPT‐HBC‐TCO_2_) were calculated as 32.39% and 34.68% respectively, at the same given data as shown in Table 1. Therefore, the thermal and exergy efficiency of the proposed combined plant was 13.21% and 13.18%, respectively, more than that of the SPT‐HBC system. The variation in the performance of the SPT‐HBC system and the proposed combined cycle plant has been shown in **Figure** **3**. The efficiencies of both systems were improved with the inlet temperature of the helium turbine. The reason for the variation in the performance with HT inlet temperature is discussed in the parametric analysis section. Apart from the thermal performance, the combined system was also compared with the SPT‐HBC system based on exergoeconomic performance. The cost of the electricity of the SPT‐HBC‐TCO_2_ system and the SPT‐HBC system were found to the 1.613 UScent kWh^−1^ and 1.642 UScent kWh^−1^ respectively at given conditions as revealed in Figure 3. The cost of electricity of the proposed (SPT‐HBC‐TCO_2_ cycle) system was lower (by ≈2%) than the SPT‐HBC system. This suggests that additional power output in the combined cycle (added power by the TCO_2_ cycle) covers the added expenditure involved by the bottoming cycle. However, the thermal and exergy efficiency of the proposed combined SPT‐HBC‐TCO_2_ cycle was around 13.21% and 13.18% higher than the SPT‐HBC system. Therefore, it can be said that the proposed SPT‐based combined cycle (HBC‐TCO_2_) is a feasible system for power generation.

### 4.2 Thermodynamic and Exergoeconomic Evaluation

The results for the present research were calculated considering the given data as shown in Table 1. Based on these input data, the thermal properties and corresponding mass flow rate at each state are calculated as shown in **Table** **7**. The thermodynamic performance for both systems, i.e., basic SPT‐HBC and the SPT‐HBC‐TCO_2_ system were computed and the results are listed in **Table** **8**. The topping cycle net power output was obtained as 14745 kW. While from the overall plant, it was found to be 16701 kW. The combined cycle(SPT‐HBC‐TCO_2_) resulted in 1946 kW additional power than that of the standalone SPT‐HBC system. This amount of power was increased due to the utilization of the waste heat by employing the bottoming TCO_2_ cycle. The total rejected heat was reduced by the addition of the TCO_2_ cycle. Around 36815 kW heat was rejected by the basic SPT‐HBC while the 34 845 kW heat was rejected by the SPT‐HBC‐TCO_2_ system. The Precooler load was reduced by 15 426 kW by the addition of the bottoming TCO_2_ cycle. Initially, the precooler load was around 19 564 kW in the SPT‐HBC system but later due to the addition of the TCO_2_ cycle it was found only as 4138 kW. It means the load of the precooler is reduced consequently the size and the cost of precooler is also reduced in the proposed novel combined system.

**Table 7:** Thermodynamic properties and mass flow rate at each state.

State	Working fluid	Mass flow rate [kg s^−1^]	Temperature [°C]	Pressure [kPa]	Enthalpy [kJ kg^−1^]	Entropy [kJ kg^−1^ °C^−1^]	Exergy [kW]
1	Helium	20.33	30	2500	34.04	−6.569	40505
2	Helium	20.33	182.6	6250	837.6	−6.353	55530
3	Helium	20.33	520	6188	2588	−3.457	73565
4	Helium	20.33	850	6064	4301	−1.61	97177
5	Helium	20.33	557.2	2577	2771	−1.401	64823
6	Helium	20.33	220	2551	1021	−4.085	45502
7	Helium	20.33	73.94	2525	262.2	−5.888	41011
8	CO_2_	66.09	180	21879	56.01	−0.7824	19138
9	CO_2_	66.09	85.38	7214	−0.1658	−0.7545	14876
10	CO_2_	66.09	30	7214	−204	−1.401	14151
11	CO_2_	66.09	63.94	21879	−177.3	−1.385	15604
12	Water	110.9	25	101.3	104.8	0.3669	0
13	Water	110.9	35	101.3	146.7	0.5049	76.09
14	Water	322	25	101.3	104.8	0.3669	0
15	Water	322	35	101.3	146.7	0.5049	221
16	Air	54.1	1125	101.3	1513	7.359	38877
17	Air	54.1	570	101.3	869.8	6.775	13488

**Table 8:** Energy results of both systems at given operating conditions.

Parameters^a)^	Units	SPT‐HBC system	SPT‐HBC‐TCO_2_ system
Thermal efficiency	%	28.61	32.39
Exergy efficiency	%	30.64	34.68
Net output power $\left({\dot{W}}_{\mathrm{net}}\right)$	kW	14754	16701
TCO_2_ cycle output power $\left({\dot{W}}_{\mathrm{net},\;\mathrm{TCO}2}\right)$	kW	‐	1946
Total heat input to the cycle$\left({\dot{Q}}_{\mathrm{Sun}}\right)$	kW	51569	51569
Total heat loss from the cycle$\left({\dot{Q}}_{\mathrm{loss}}\right)$	kW	36815	34845
Heat load on the precooler $\left({\dot{Q}}_{\mathrm{precooler}}\right)$	kW	19564	4138
Power output from the helium turbine $\left({\dot{W}}_{\mathrm{HT}}\right)$	kW	31089	31089
Power output from the TCO_2_ turbine $\left({\dot{W}}_{\mathrm{TCT}}\right)$	kW	‐	3713

^a)^ DNI = 850 W/m^2^, CPR = 2.5, T_8_ = 160 °C, T_4_ = 850 °C

Further exergy evaluation of this system is conducted to investigate the weak points. Exergy destruction, exergy destruction ratios, and exergy efficiency are the parameters to be investigated. At the given input condition, the exergetic results are listed in **Table** **9**. Exergy from the sun (48 152 kW) has been considered as the input exergy to both systems. A lot of exergy destruction occurs in both systems. To find the position where the exergy destruction occurs (in depth analysis) has been done in this section. The exergy efficiency of the basic SPT‐HBC system and overall plant (SPT‐HBC‐TCO_2_) were found as 30.64% and 34.68%, respectively. Total exergy output from the proposed combined system was increased due to the extra amount of work produced by the bottoming TCO_2_ cycle. Therefore, the proposed system's exergy efficiency is greater than the basic system.

**Table 9:** Exergetic results of both systems at given operating conditions.

Parameters^a)^	Units	SPT‐HBC system	SPT‐HBC‐TCO_2_ system
Total exergy input $\left({\overset{\cdot }{\mathrm{EX}}}_{\mathrm{Sun}}\right)$	kW	48152	48152
Total exergy output $\left({\dot{W}}_{\mathrm{net}}\right)$	kW	14754	16701
Total exergy destruction $\left({\overset{\cdot }{\mathrm{EXD}}}_{\mathrm{Total}}\right)$	kW	33398	31451
Exergy efficiency	%	30.64	34.68

^a)^ DNI = 850 W/m^2^, CPR = 2.5, T_8_ = 180 °C, T_4_ = 850 °C

Exergy efficiency and exergy destruction for each component were found to investigate the weak point based on exergetic analysis. The detailed exergetic results of the proposed combined system (SPT‐HBC‐TCO_2_) are shown in **Table** **10**. Exergy evaluation reveals that the highest exergy destruction was found in the heliostat field because solar irradiation is a high‐quality energy with a heat source of high temperature around 4500 K.^[^ ^52^, ^53^ ^]^ Since this radiation is received by the receiver at a temperature range of 1200–1500 °C, there is a substantial irreversibility. The exergy efficiency of the heliostat was considered as 75%. As the combustion process does not occur in the IHE (the primary reason for irreversibility in traditional power systems), also there is no much difference in temperature between the helium and air (HTF) in the IHE. Due to these reasons, the power generation unit (HBC‐TCO_2_ cycle) has a high exergy efficiency of ≈65.78%. However, the power plant's total exergy efficiency is quite low due to the significant exergy destructions in the heliostat field and receiver. The component‐wise exergy parameters were also calculated and listed in Table 10. Overall power plant's highest and lowest exergy destruction components were heliostats (12 038 kW) and pumps (313.5 kW) respectively. Heliostat contributed alone 38.27% of the plant's total exergy destruction. Also, the exergy destruction ratio was found as 25%. It means if the heliostat is designed properly, exergetic performance may be increased by 25%. After the heliostat, the second most exergy destruction component is the receiver (10 725 kW). It contributed around 34.10% of the plant's total exergy destruction. Its exergy efficiency was found as 70.30%. Its exergy destruction ratio was found as 29.7%. It means 29.7% of total inlet exergy was destructed in the receiver. If the receiver is carefully designed, the performance can be enhanced. Among the different components, the helium turbine contributed (96.09%) the highest exergy efficiency. The reason is that the difference in temperature between the fuel and product exergy of the turbine was quite low. That's why low exergy destruction has occurred in the turbine. Its exergy destruction ratio was ≈3.91% only. It means further helium turbine has less potential to enhance the exergetic performance. It was seen that the precooler has the lowest exergy efficiency (15.03%). This much amount of exergy is not destruction however it is the form of exergy loss through the cooling water that is flowing in the precooler. The performance of the system can be further improved if this exergy is utilized by providing the other low‐temperature power cycle.

**Table 10:** Detailed exergy results for the proposed SPT‐HBC‐TCO_2_ system at given operating conditions.

Components	${\overset{\cdot }{\mathrm{EX}}}_{\mathrm{fuel}}$ [kW]	${\overset{\cdot }{\mathrm{EX}}}_{\mathrm{product}}$ [kW]	$\overset{\cdot }{\mathrm{EXD}}$ [kW]	η_ex_[%]	y_D_[%]
Heliostat	48152	36114	12038	75	25
Receiver	36114	25389	10725	70.30	29.7
IHX	10447.05	8671	1776	93	17
HT	32352.94	31087.94	1265	96.09	3.91
Recuperator	19309.30	18023.3	1286	93.34	6.66
WHRU	4496.24	3539.54	956.7	78.70	21.3
Precooler	506.17	76.07	430.1	15.03	84.97
HC	15733.17	14424.17	1309	91.68	8.32
TCT	4263.36	3712.96	550.4	87.09	12.91
Condenser	724.42	221.02	503.4	30.51	69.49
Pump	1766.19	1452.69	313.5	82.25	17.75

**Table** **11** outlines the exergoeconomic evaluation results of the different elements of the proposed SPT‐HBC‐TCO_2_ system. The greatest value of the ${\dot{C}}_{D}+\dot{Z}$ is obtained by the heliostats. It indicates that special attention is needed to design the heliostat to enhance the system's exergoeconomic performance. The large value of 80% for the exergoeconomic factor indicate that investment cost, operation, and management cost $\left(\dot{Z}\right)$ dominates over the cost rate of exergy destruction. The second greatest value of the ${\dot{C}}_{D}+\dot{Z}$ was obtained by the receiver around 164.736 $ h^−1^ with an exergoeconomic factor of 52.53%. However in the power generation unit (HBC‐TCO_2_), the highest value of the ${\dot{C}}_{D}+\dot{Z}$ was obtained by the TCO_2_ turbine ≈34.3764 $ h^−1^. Its exergoeconomic factor is 75.01% and its exergy destruction ratio is 12.91%. Therefore, this component is satisfied based on the exergy and exergoeconomic performance point of view.

**Table 11:** Exergoeconomic results of the proposed SPT‐HBC‐TCO_2_ system.

Components	${\dot{C}}_{D}$ [$ h^−1^]	$\dot{Z}$ [$ h^−1^]	${\dot{C}}_{D}+\dot{Z}$ [$ h^−1^]	f [%]
Heliostat	35.4780	141.9120	177.3900	80
Receiver	78.1920	86.5440	164.7360	52.53
IHE	15.9840	0.2502	16.2300	1.54
HT	12.2580	5.1624	17.4200	29.63
Recuperator	12.4560	1.4896	13.9456	10.68
WHRU	11.4588	1.3698	12.8286	10.67
Precooler	4.0572	0.5662	4.6234	12.24
HC	11.1996	2.0487	13.2483	15.46
TCT	8.5896	25.7868	34.3764	75.01
Condenser	7.8588	0.7995	8.6583	9.23
Pump	2.5632	8.2332	10.7964	76.25
Overall plant	200.0952	274.176	474.2712	57.80

Examining the exergoeconomic factor for the IHE, it was found as the lowest (1.54%) among the other components. It indicates that 98.46% of the cost is because of exergy destruction. This shows that almost complete cost is due to the cost of exergy destruction. Therefore, by reducing the exergy destruction in the IHE, system performance can be enhanced. The exergy destruction in IHX can be reduced by increasing the heat transfer area which eventually increases the area and the cost of heat exchanger. A similar discussion for the recuperator and WHRU may be done. Since both have low exergoeconomic factor values.

A lower value of the exergoeconomic factor of the helium compressor (HC) reveals that high capital cost is preferable to improve the compressor pressure ratio. Exergoeconomic factor for the HT is 29.63%. It indicates that 69.37% of the total cost is because of exergy destruction. After reducing the exergy destruction cost of the component, exergoeconomic performance is improved. However, HT has the highest exergy efficiency around 96.03%. Therefore, its exergy performance is satisfactory.

The exergoeconomic factor value for the overall proposed SPT–HBC‐TCO_2_ system was calculated as 57.80%. This indicates that 42.2% of the total cost is related to the exergy destruction. So, it can be said that by reducing the component exergy destruction, exergoeconomic performance can be enhanced.

### 4.3 Parametric Analysis

Parametric assessment is necessary to examine the influence of the independent variables such as helium Turbine inlet temperature, pressure ratio of compressor (CPR),turbine inlet temperature of TCO_2_, pump pressure ratio (PPR), solar intensity (DNI), efficiency of receiver, heliostat field efficiency on the system's thermodynamic and the exergoeconomic performance. The impact of variation of particular parameter has been studied by maintaining the values of other variables constant as mentioned in Table 1.

**Figure 4:**
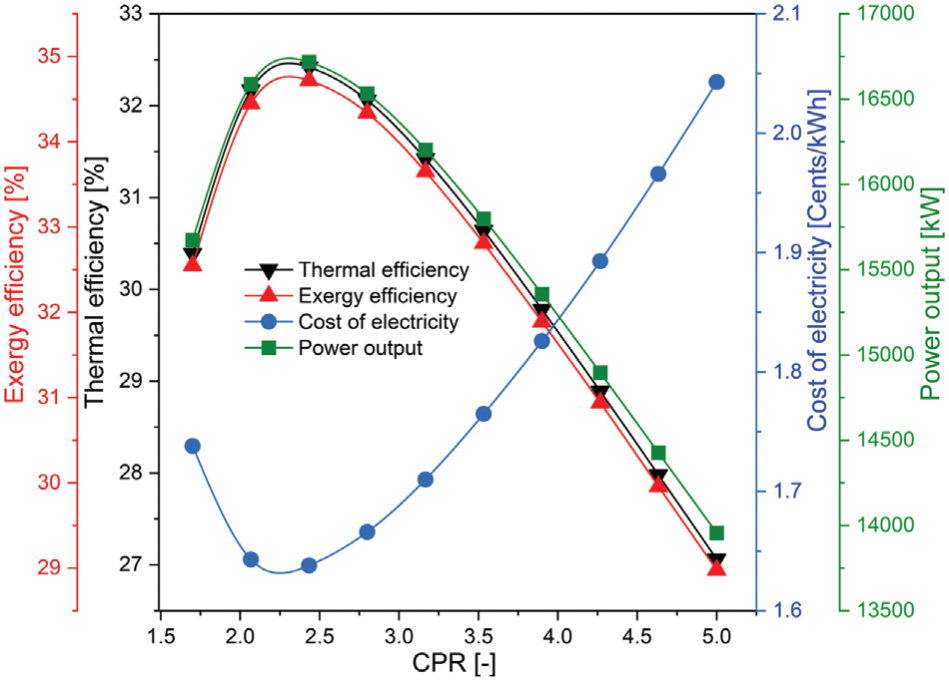
Variation of performance of the proposed SPT‐HBC‐TCO_2_ system with the CPR.

**Figure** **4** describes the variation in the performance of the overall system with CPR. For the helium Brayton cycle, the CPR is the critical parameter whose implications require discussion. The overall combined system (SPT‐HBC‐TCO_2_ cycle) performance was evaluated with varying CPR; as the CPR increases, efficiencies first increases and reaches a maximum of 2.43 CPR and then decreases continuously. The highest thermal and exergy efficiency were found as 32.42% and 34.72% respectively at 2.43 of CPR and then decreases gradually. This pattern can be explained as the CPR increases the compression work and the expansion will also increase, however during this period the rate of improvement of expansion work dominates over the the rate of improvement in compression work till CPR of 2.43 where the maximum work output is achieved. That leads to improvement in the combined cycle's efficiencies. After the CPR of 2.43 results were shown vice versa. Similar to the efficiencies the power output also has the same pattern. Net power output first increases and then decreases continuously. Maximum output power was found at 2.43 of CPR with a value of 16718 kW. Apart from the thermal performance, the electricity cost of the proposed plant has the opposite trend to the thermal performance. First, it decreases and then it increases with the varying CPR. This trend can be explained by Equation (24) where the net work output is in the denominator. However, the trend work output is already explained. Therefore, cost of electricity has an opposite trend to work output. The lowest value cost of electricity is preferable which is found as 1.638 UScent kWh^−1^ at 2.43 of CPR.

**Figure 5:**
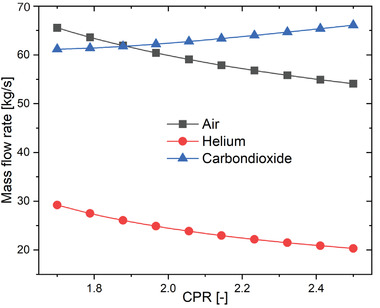
Mass flow rate variation with CPR.

**Figure** **5** reveals the mass flow rate variation with CPR. CPR also influences the working fluids' mass flow rate in different sub‐cycles, i.e., mass flow rate of air in the SPT system through the receiver, helium in the topping cycle, and carbon dioxide in the TCO_2_ cycle. Keeping constant the input variables values as discussed, the helium flow rate and the air flow rate decreases with the increasing CPR while the mass flow rate of carbon dioxide increases with CPR. It can be interpreted as the CPR increases the pressure inside the topping cycle is at the higher pressure side therefore less mass of helium flow is required since the work output is constant for the topping cycle. Now air mass flow rate in the SPT system also decreases with the increasing CPR because of the same reason as above discussed. The mass flow rate of carbon dioxide increases with the CPR because the amount of heat exchange in the WHRU increases due to high operating pressure in the topping cycle. The mass flow rate of air, helium, and carbon dioxide varies from 57.45 to 37.56 kg s^−1^, 31.54 to 14.34 kg s^−1^, and 52.34 to 74.76 kg s^−1^, respectively as CPR increased from 1.6 to 2.6 as given in Figure 5.

**Figure 6:**
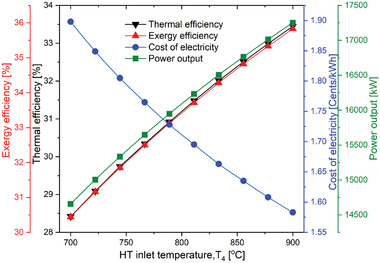
Performance variation of the proposed SPT‐HBC‐TCO_2_ system with HTIT.

**Figure** **6** depicts the effect of HT Inlet Temperature (HTIT) on the overall system performance. It can be observed from the graph that as the temperature increases from 700 to 900 °C, the thermal efficiency, exergy efficiency and the net output power of the proposed overall plant also increases from 28.42% to 33.45%, 30.44% to 35.83%, and 14656 to 17251 Kw, respectively. This is because as the HTIT increases the enthalpy difference across the turbine increases due to which the expansion work increases that leads to an increment of net power output consequently increasing the thermal and exergy efficiencies. This variation was performed at optimum CPR of 2.43, 850 W m^−2^ of DNI, and 180 °C inlet temperature of the TCO_2_ cycle. However, the electricity cost decreased continuously with the increasing HTIT. An increase in the net work output would lead to a lower cost of electricity. Electricity cost varied from 1.898 UScent kWh^−1^ to 1.583 UScent kWh^−1^ with a variation of HTIT from 700 to 900 °C.

**Figure 7:**
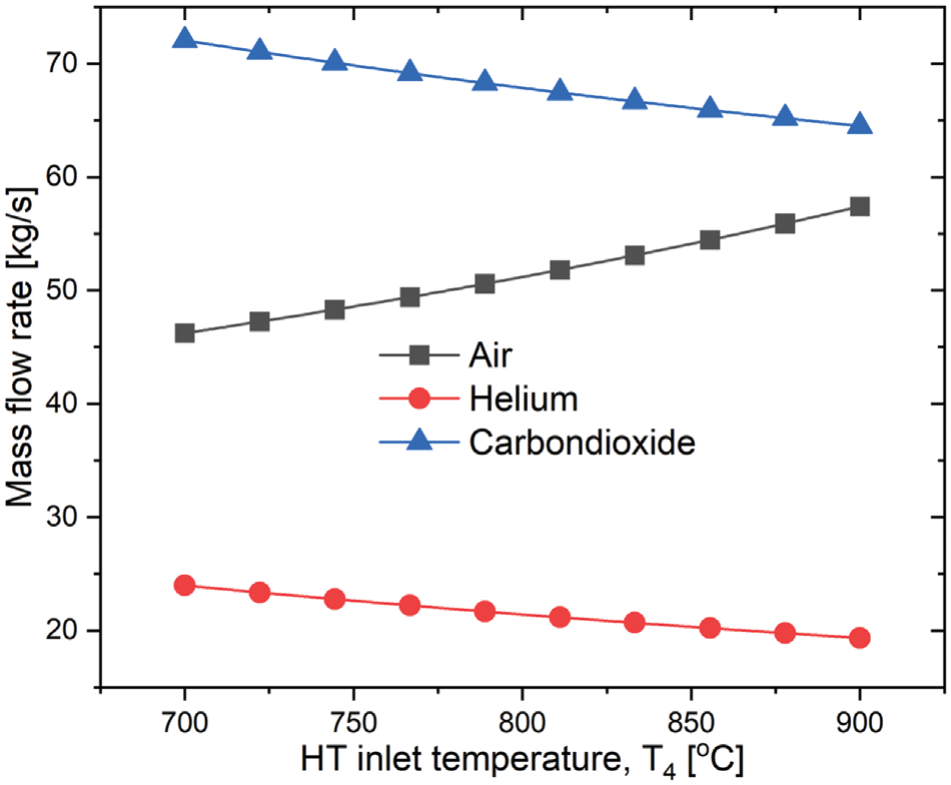
Mass flow rate variations with HTIT.

**Figure 8:**
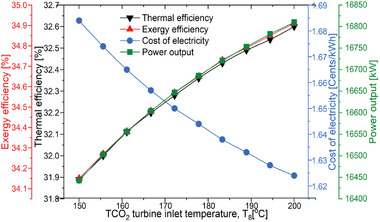
Performance variation of the proposed SPT‐HBC‐TCO_2_ system with TCT inlet temperature.

Apart from the thermal performance, HTIT also affects the flow rate of the working fluid in the considered power plant. The airflow rate in the SPT subsystem enhanced while the mass flow rate of the helium and the carbon dioxide decreased with HTIT as seen in Figure 8. The mass flow rate of air increases since the inlet temperature of air entering IHE is fixed (1125 °C) while the heat load in IHE increases as the HTIT increases. The mass flow rate of helium and carbon dioxide decreases because as the temperature increases the density of working fluid decreases since the work output remains constant. Air, helium, and carbon dioxide mass rates varies from 41.63 to 59.57 kg s^−1^, 21.92 to 16.14 kg s^−1^, and 59.69 to 52.02 kg s^−1^, respectively, as HTIT increased from 700 to 900 °C as illustrated in **Figure** **7**.

The impact of the TCO_2_ turbine inlet temperature of on system performance is discussed in this section. The overall efficiency of the plant was marginally affected by the temperature inlet to the TCO_2_ turbine. Net output power output affected more than thermal efficiencies. The reason is that the net output power of the combined cycle also includes the power output by the bottoming cycle. The contribution of the power generated by bottoming cycle during the turbine inlet temperature variation is much less than the net combined cycle power. Therefore, effect on the efficiency is marginal. As the temperature is enhanced from 150 to 200 °C, thermal and exergy efficiencies are improved by only 2.05% and 2% for the overall power plant performance as seen in **Figure** **8**.

The cost of electricity is decreased with the TCO_2_ turbine inlet temperature as the work output increases. The cost decreases from 1.684 UScent kWh^−1^ to 1.624 UScent kWh^−1^ with an increase in temperature of 150 to 200 °C as illustrated in Figure 8. It was observed that cost was also marginally affected by the temperature. Since the bottoming cycle is a low‐temperature cycle therefore low capital and exergy destruction cost are associated with it. It can be said that the cost of electricity is not very sensitive to the bottoming cycle performance.

**Figure 9:**
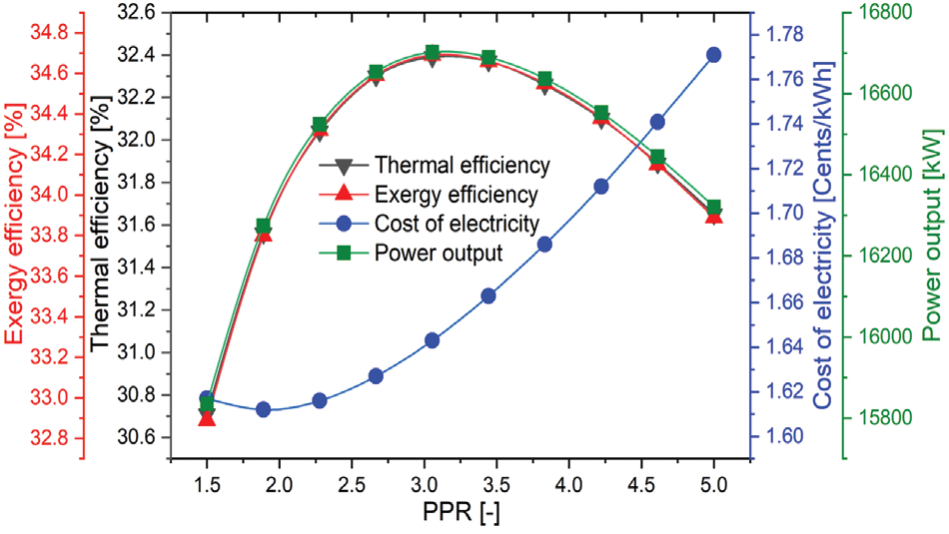
Performance variation of the proposed SPT‐HBC‐TCO_2_ system with PPR.

**Figure** **9** depicts the effect of pump pressure ratio (PPR) on the thermal efficiency, exergy efficiency, and cost of electricity generation for the combined system. The graph illustrates how the thermal and exergy efficiency of the system increases first, reaches a maximum value, and then gradually decreases as the PPR increases. This is because the thermal and exergy efficiency increases until the work produced by the TCO_2_ turbine dominates and starts decreasing when the amount of work consumed by the pump increases. As a result, the optimum value of the PPR was obtained to be 3.056. The highest value work, exergy, and thermal efficiency of the overall power plant were observed as 16702 kW, 34.69%, and 32.39% respectively at 3.056 of PPR.

The cost of electricity initially decreases with the increase in PPR attains a minimum value and then increases continuously as the amount of work consumed by the pump increases the cost of electricity for power supply to the pump also increases. As it can be observed from the Figure 9, the variation in PPR from 1.5 to 1.889 reflects the decrease in electricity cost from 1.612 to 1.771 UScent kWh^−1^ while the cost of electricity increases from 1.612 to 1.771 UScent kWh^−1^ with an increase in PPR from 1.889 to 5.

**Figure 10:**
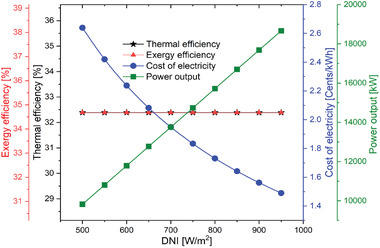
Performance variation of the proposed SPT‐HBC‐TCO_2_ system with DNI.

The effects of the solar parameter such as DNI, heliostat efficiency, and receiver efficiency are discussed further in this section. Solar radiation is another significant parameter that affects the thermal and economic performance of the power generation unit. As observed in **Figure** **10**, the higher power output is obtained at a higher value of DNI. This is because a significant amount of energy is available at the heliostatic field as the DNI increases. Since the available input heat increases at the receiver, the mass flow rate of the HTF fluid increases due to which the net output power of overall system increases. However, efficiencies with DNI are constant since there is simultaneous increment in both numerator and denominator as observed in Equations (8) and (9).

On the other side the economic performance, Figure 10 reveals that the lower value of the electricity cost is obtained at the higher value of the DNI. It implies that increments in net power output can compensate for the increments in investment cost by increasing the size of the component. As it can be seen, that combined cycle power was increased from 9824 to 18665 kW with an increment in the DNI from 500 to 950 W m^−2^. This value accounted for 100% increment in power output. Also, the cost of electricity decreases from 2.639 to 1.49 UScent kWh^−1^, if the DNI increases from 500 to 950 W m^−2^.

**Figure 11:**
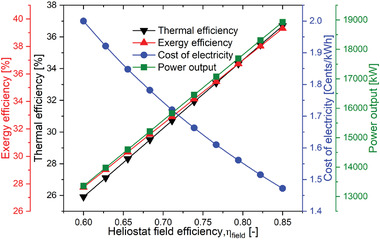
Performance variation of the proposed SPT‐HBC‐TCO_2_ system with η_field_.

In order to comprehend the influence on system performance and design, it is essential to understand the impact of solar subsection factors on the performance of the whole system. In the current examination the receiver efficiency (η_receiver_) and heliostat field efficiency (η_field_) are considered as solar sub‐section parameters. As **Figure** **11** depicts the net power output, exergy efficiency, and thermal efficiency increases with the increase in heliostat filed efficiency (η_field_) because the heat loss from the heliostat decreases consequently additional heat energy is received by the receiver. Due to which the HTF mass flow rate in the receiver increases and then the helium flow rate increases in the IHX during the heat exchange. This leads to improvement in the overall net output power output. Since exergy and thermal efficiency are directly related to the amount of power generated, both were enhanced. However, the cost of electricity has an inverse relation with the field efficiency due to its inverse relationship with the net power output. As field efficiency increases from 0.6 to 0.85, the cost of electricity decreases from 2 to 1.472 UScent kWh^−1^ as shown in Figure 11.

**Figure 12:**
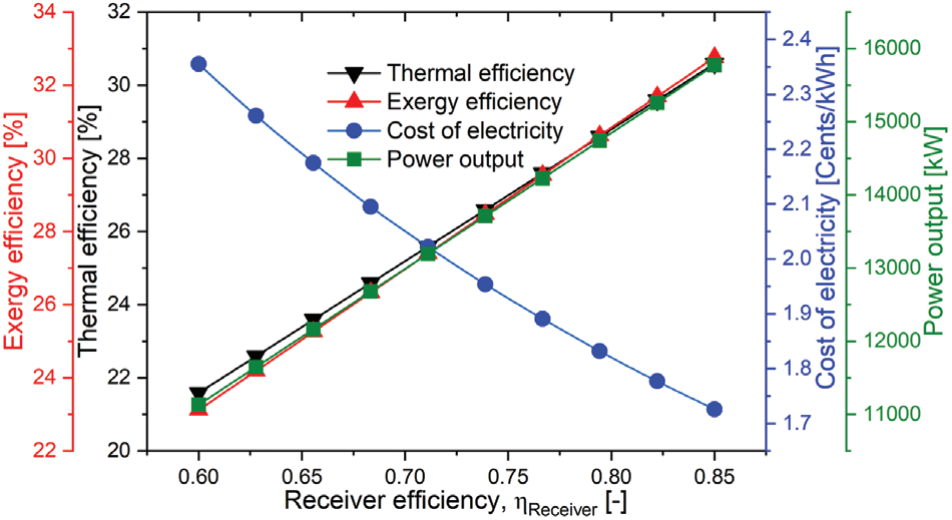
Performance variation of the proposed SPT‐HBC‐TCO_2_ system with η_receiver_.

In addition to the field efficiency, the efficiency of the receiver is also a crucial parameter of the solar sub‐system that influence the thermodynamic and economic performance of the combined system. The thermal performance of overall power plant is improved, as shown in **Figure** **12**, when the receiver efficiency (η_receiver_) is increased. Due to the increase in the receiver efficiency (η_receiver_) heat loss to the environment decreases. Therefore, the amount heat energy received by the receiver will increase. This results in advancement in the mass flow rate of the HTF and helium as explained previously. Consequently, the net power output would enhance. As a result of the same heliostat area, the plant's overall energy intake remains unchanged due to which the exergy efficiency increases and the cost of electricity decreases. It can be observed that the cost of electricity decreases by 3.95% on average for each 4% increase in receiver efficiency.

### 4.4 Comparison with Previous Studies

As discussed previously, numerous irreversibilities in the solar sub‐section of any SPT plants arises and cannot be prevented. Therefore, using a reliable power generation unit is essential for enhancing the overall performance of the SPT plant. In this respect, the performance of the current combined system based on SPT is compared to that of preceding systems published by other researchers. In order to perfom an authentic comparative study, similar solar conditions are taken into the consideration as published by the previous authors. **Table** **12** depicts the comparative study between the outcome of the present study and the previously published work.

**Table 12:** Results comparison with previous studies.

Systems	DNI [kW m^−2^]	η_field_	η_receiver_	η_field_ × η_receiver_	η_PGU_[%]	η_th,overall_[%]	η_ex,overall_[%]
Rankine cycle (regenerative)^[^ ^66^ ^]^	0.8	0.75	0.9	–	37.9	22.9	24.5
Supercritical Rankine cycle (regenerative)^[^ ^66^ ^]^	0.8	0.75	0.9	–	42.1	25.7	27.4
Proposed plant	0.8	0.75	0.9	–	47.19	32.09	34.21
SCO_2_ cycle^[^ ^67^ ^]^	1	N.A.	N.A	0.62	42.48	26.23	N.A
Combined TCO_2_‐ORC^[^ ^67^ ^]^	1	N.A	N.A	0.62	43.96	27.14	N.A
Proposed plant	1	–	–	0.62	50.32	31.89	34.02

The results demonstrate that the PGU proposed in the present study outperforms prior similar systems. As it can be observed, compared to the SPT‐based Rankine system and the SPT‐based sCO_2_ system, the SPT‐HBC‐TCO_2_ system employed in this study achieves greater overall efficiency. From an economic perspective, the proposed system in this paper has an acceptable cost of electricity in comparison to other hypothetical or operational plants. Recently, Abdelhady^[^ ^68^ ^]^ revealed 50 MW solar power plant has a value of 13.38 UScent kWh^−1^ of electricity for the construction of the solar dish technique. This plant developed in the western desert of Egypt where it absorbs energy at high solar radiation in the planet. Also, the acquired cost of power for this system is relatively similar to the lowest values for the already available CSP‐photovoltaic hybrid solar plants, which are stated to be in the range of 0.074–0.113 $ kWh^−1^.^[^ ^69^ ^]^

## 5 Conclusions

In the current work, a novel helium‐based supercritical Brayton cycle combined power generating system for solar power tower plants is developed. In order to utilize the waste heat from helium Brayton cycle, transcritical carbon dioxide cycle is combined to generate additional power. To determine how sensitive the performance parameters were to the plant's independent factors, a parametric analysis was done. The results discussed that generated extra power by waste heat cycle balances the induced expenditure. Under standard operating conditions, it is discovered that the SPT‐based combined cycle (SPT‐HBC‐TCO_2_ cycle) produces a thermal efficiency of 32.39% and exergy efficiency of 34.68% with an electricity cost of 1.613 UScent kWh^−1^. Thermal and exergy efficiency values are better by 13.21%, and 13.18%, respectively, however the cost of electricity is ≈2% lowered, than the comparable values for the standalone system (SPT‐HBC), demonstrating a significant performance increase from waste heat recovery. The combined cycle's HBC system, with a value of 58.11%, has the highest exergy efficiency of all the subsystems (SPT, HBC, and TCO_2_ cycle). Although, the power generation unit (HBC‐TCO_2_ cycle)’s exergy efficiency was measured as 65.78%. Also, the solar sub‐system has the highest exergy destruction rate, and cost rate around 72.37% and 56.8% respectively, of the proposed overall plant. A comparison with previous studies based on sCO_2_ and Rankine‐based power plants shows that the SPT‐based proposed combined cycle in the present research offers higher exergy efficiency and thermal efficiency.

## Conflict of Interest

The authors declare no conflict of interest.

## Data Availability

The data that support the findings of this study are available from the corresponding author upon reasonable request.
